# TolC Promotes ExPEC Biofilm Formation and Curli Production in Response to Medium Osmolarity

**DOI:** 10.1155/2014/574274

**Published:** 2014-08-27

**Authors:** Bo Hou, Xian-Rong Meng, Li-Yuan Zhang, Chen Tan, Hui Jin, Rui Zhou, Jian-Feng Gao, Bin Wu, Zi-Li Li, Mei Liu, Huan-Chun Chen, Ding-Ren Bi, Shao-Wen Li

**Affiliations:** State Key Laboratory of Agricultural Microbiology, College of Veterinary Medicine, Huazhong Agricultural University, No. 1 Shizishan Street, Wuhan, Hubei 430070, China

## Abstract

While a high osmolarity medium activates Cpx signaling and causes CpxR to repress *csgD* expression, and efflux protein TolC protein plays an important role in biofilm formation in* Escherichia coli,* whether TolC also responds to an osmolarity change to regulate biofilm formation in extraintestinal pathogenic* E. coli* (ExPEC) remains unknown. In this study, we constructed* ΔtolC* mutant and complement ExPEC strains to investigate the role of TolC in the retention of biofilm formation and curli production capability under different osmotic conditions. The* ΔtolC* mutant showed significantly decreased biofilm formation and lost the ability to produce curli fimbriae compared to its parent ExPEC strain PPECC42 when cultured in M9 medium or 1/2 M9 medium of increased osmolarity with NaCl or sucrose at 28°C. However, biofilm formation and curli production levels were restored to wild-type levels in the* ΔtolC* mutant in 1/2 M9 medium. We propose for the first time that TolC protein is able to form biofilm even under high osmotic stress. Our findings reveal an interplay between the role of TolC in ExPEC biofilm formation and the osmolarity of the surrounding environment, thus providing guidance for the development of a treatment for ExPEC biofilm formation.

## 1. Introduction

One function of bacteria is the formation of biofilms composed of a bacterial community on a surface, leading to a persistent infection in both animals and humans [1, 2]. The amount of antibiotics or disinfectants necessary to kill bacteria in a biofilm can be up to 1,000 times greater than that in corresponding planktonic cultures [3] and thus, it is difficult for a biofilm to be eradicated. Extraintestinal pathogenic* Escherichia coli* (ExPEC) strains are pathogens that cause a variety of clinical syndromes, including urinary tract, central nervous, circulatory, and respiratory system infections in humans and animals. Moreover, animals can be reservoirs for these ExPEC strains, which can then be transmitted to humans [4, 5]. For example, in our previous studies, ExPEC isolates detected in 315/3127 (10.1%) pigs among 19 provinces of China exhibited high multidrug resistance (MDR) [6, 7]. One of the main reasons for the threat of ExPEC strains to human health is biofilm formation by ExPEC strains. As such, uropathogenic* Escherichia coli* (UPEC), a type of ExPEC strain that forms biofilms, causes urinary tract infection that is hard to eradicate with antibiotics [8].

Thus, it is essential to understand the factors that influence biofilm formation in order to find an effective way to prevent biofilm formation and destroy a biofilm. TolC belongs to the outer membrane efflux protein (OEP) family of* E. coli* and plays important roles in maintaining the structure and function of the outer membrane [9, 10]. Some previous studies demonstrated that some* tolC* mutants without TolC expression are tolerant of colicin E1 [11] and hypersensitive to certain dyes, drugs, and detergents [12]; have altered bacterial virulence [13]; and pump the biomolecules derived from bacterial self-metabolism [14, 15]. Recently, there has been an increasing interest in the correlation between efflux proteins, including TolC, and biofilm formation. The addition of efflux pump inhibitors (EPIs) may reduce or abolish biofilm formation by* E. coli* and* Klebsiella* [16]. Mutants, such as* E. coli* K-12 strain, that lack functional multidrug efflux pump-related genes such as* emrD, emrE, emrK acrD, acrE*, or* mdtE* exhibit decreased biofilm formation [17]. Moreover,* Salmonella typhimurium* lacking functional TolC loses the ability to form biofilms [18]. Meanwhile, in* E. coli*, the CpxA-CpxR signaling together with the sigma (E) and sigma (32) signal pathways regulates gene expression in response to adverse conditions. A high osmolarity medium activates Cpx signaling and causes CpxR to repress* csgD* expression, an important event in regulating curli and cellulose production [19]. However, while it is recognized that efflux protein TolC protein plays important roles in* E. coli* K-12 strain and* S. typhimurium* biofilm formation, whether TolC also responds to changes in osmolarity to regulate biofilm formation in ExPEC stains remains unknown.

Therefore, the objective of this study was to determine whether TolC plays an essential role in biofilm formation of an ExPEC strain in response to different osmolarity conditions using a* ΔtolC* mutant strain under different osmotic conditions. Because curli fimbriae are the major component of the extracellular matrix involved in* E. coli* biofilm formation [20, 21], we investigated the effect of the* ΔtolC* mutation on curli production and curli biosynthesis-related gene expression under different osmolarity conditions.

## 2. Materials and Methods

### 2.1. Bacterial Strains, Plasmids, and Growth Media

A wild-type (WT) parent ExPEC strain PPECC42, which is highly pathogenic in mice and pigs and belongs to serotype O_11_, was isolated from the lung of a pig in Hunan Province of China in 2006. Plasmid* pRE112* was used as a suicide vector for homologous recombination to construct the* ΔtolC* mutant.* E. coli *χ*7213* was a host for* pRE112* in the conjugal transfer [22].* E. coli* DH5*α* and plasmid* pHSG396* were purchased from Takara Bio (Japan). All strains were routinely cultivated in Luria-bertani (LB) medium supplemented with 100 *μ*g mL^−1^ampicillin and 50 or 25 *μ*g mL^−1^ chloramphenicol and in M9 medium or 1/2 M9 medium, a low-osmolarity minimal medium prepared by dilution of M9 medium with an equal amount of water (Table 1).

### 2.2. Construction of *ΔtolC* Mutant and Its Complement Strain

The upstream and downstream regions of* tolC* gene were amplified by PCR from genomic DNAs of ExPEC strain PPECC42 with primers P1/P2 and P3/P4, respectively (Table 2). The purified upstream and downstream PCR products were mixed, and the overlapping PCR was performed. The primers P1/P4 were used again in order to amplify the disrupted* tolC* gene with a 158-bp fragment deleted in the* tolC* open reading frame (ORF). The plasmid* pREΔtolC* with the disrupted* tolC* gene was introduced into* E. coli *χ*7213.* The WT strain was cocultured with the *χ7213*-Δ*tolC strain*. Transformants resistant to chloramphenicol and sensitive to sucrose were selected on LB agar media containing chloramphenicol and sucrose and grown on LB agar media without NaCl or antibiotics to select a strain resistant to sucrose and sensitive to chloramphenicol. The final PPECC42* ΔtolC* strain that lacked TolC expression was confirmed by PCR using the primers che-U/che-D (Table 2).

To rescue TolC expression in the* ΔtolC* mutant, the complete coding region of the* tolC* gene was amplified from the genomic DNAs of the WT strain by PCR using the primers clo-U/clo-D (Table 2) and inserted into* pHSG396* to generate the plasmid* pHSG396-tolC*, which was then introduced into the* ΔtolC* strain using electrotransformation instruments (Bio-Rad, USA). The rescued Cm-*tolC* with the full-length tolC gene were selected on LB agar medium containing chloramphenicol and confirmed by PCR using primers che-U/che-D.

### 2.3. Minimal Inhibitory Concentration (MIC) Determinations

To confirm the construction of* ΔtolC* mutant and its complement strain, antimicrobial MICs to the strain were determined on Mueller-Hinton medium (Hopebio Bio., Qingdao, China) by the standard broth doubling microdilution method according to the Clinical and Laboratory Standards Institute Guidelines [25]. The MICs were defined as the lowest concentration that completely inhibited visible growth after incubation at 37°C for 18 h. The following antimicrobials were used: amikacin, gentamycin, erythromycin, streptomycin, ampicillin, florfenicol, norfloxacin, chloramphenicol, tetracycline, ciprofloxacin, and sodium dodecyl sulfonate (SDS). Samples were assayed in triplicate, and each assay was performed at least three times.

### 2.4. Determination of Growth Kinetics

The 1 : 100 diluted overnight cultures were cultured in M9 or 1/2 M9 medium at 28°C with shaking at 200 rpm. Samples were taken hourly, and the optical densities were measured at 600 nm (OD_600_) using a BioPhotometer (Eppendorf, Germany). The data acquired were from three independent experiments, with each sample measured in triplicate.

### 2.5. Crystal Violet Biofilm Assay

Biofilm formation was evaluated by a crystal violet assay according to the method described previously [26]. Overnight cultures in LB media were diluted 1 : 100 in fresh M9 or 1/2 M9 medium to an OD_600_ of 0.1. Diluted suspensions were placed in a flat-bottomed 96-well polystyrene microtiter plate (Nunc, Denmark) and incubated at 28°C and 100% humidity without shaking. After 2 or 5 days, the media were removed, and the wells were gently washed with sterile distilled water to remove any unbound cells. Biofilms in each well were stained with 200 *μ*L of 1% crystal violet for 15 min. Crystal violet was removed, and each well was washed with sterile distilled water to remove any unbound dye. The stained biofilm was solubilized with 125 *μ*L of 33% acetic acid, and the OD was measured at 630 nm (OD_630_) using a Universal Microplate Reader (Bio-Tek, USA) to quantify the total biofilm mass. All biofilm assays were performed three times with samples assayed in octuplicate. The strains were cultured in M9 or 1/2 M9 medium at 28°C because the WT strain could not form a biofilm in M9 or 1/2 M9 medium at 37°C or in LB medium at 28°C or 37°C (data not shown).

Based on the OD_630_ values of the bacterial biofilms, strains were classified according to the method described previously [26]. Briefly, the cutoff OD (ODc) was defined as three standard deviations above the mean OD of the negative control. Strains were classified as follows: OD < ODc = no biofilm production; ODc < OD < 2 × ODc = weak biofilm producer; 2 × ODc < OD < 4 × ODc = moderate biofilm producer; and OD > 4 × ODc = strong biofilm producer.

### 2.6. Scanning Electron Microscopy (SEM) of Biofilms

Strains were cultured on M9 medium or 1/2 M9 medium at 28°C for 5 days using the same procedures as described for the biofilm assay. Coverslips were immersed in the media in 24-well plates. The adhered bacteria were washed three times with phosphate-buffered saline (PBS), fixed in 2.5% glutaraldehyde, and dehydrated in a series of ethanol dilutions (30%, 50%, 70%, 80%, 95%, and 100%). The samples were then soaked in isoamyl acetate, critical point dried with CO_2_, and coated with gold alloy. The prepared samples were viewed and photographed using a JSM-6390 scanning electron microscope (JEOL, Japan).

### 2.7. Effects of Medium Solutes on the Role of TolC in Biofilm Formation

In order to determine if the role of TolC in biofilm formation is related to the osmolarity of the medium, a 2-fold serially diluted NaCl or sucrose solution was added to the 1/2 M9 medium to change the medium osmolarity according to the method described previously [19]. Biofilm formation in different media was assessed for both WT and* ΔtolC* mutant strains using the crystal violet biofilm assay.

### 2.8. Staining of Curli Fimbriae

Phenotypic differences in curli expression were visualized on M9 or 1/2 M9 agar plates containing 40 *μ*g mL^−1^ Congo red (Amresco, USA) and 20 *μ*g mL^−1^ Coomassie brilliant blue (Solarbio, China) according to the method described previously [27]. One- microliter aliquots of overnight cultures of each bacterial strain grown in LB medium were streaked onto CR agar plates and incubated at 28°C for 5 days. Strains grown on the CR agar plates were divided into four morphotypes [28, 29]:* rdar* (red, dry, and rough; curli and cellulose),* pdar* (pink, dry, and rough; cellulose only),* bdar* (brown, dry, and rough; curli only), and* saw* (smooth and white; neither curli nor cellulose). The experiments were repeated three times. Comparisons of colonies were made between the WT and mutant strains grown on the same plates.

### 2.9. Quantitative Reverse Transcriptase PCR (qRT-PCR) of *rrsG*, *csgD*, and *csgB* Genes

In order to determine whether the role of TolC in curli production is at the transcriptional or assembly level, we used real-time quantitative RT-PCR to determine the expression of* csgB*, the gene encoding the structural component of curli [20], and* csgD*, a transcriptional regulator [30, 31] that forms a regulatory cascade along with sigma factors RpoS-RpoE for activation of curli gene expression and biofilm production [20]. Total RNAs of the WT and* ΔtolC* mutant strains grown at 28°C for 1, 3, or 5 days were extracted using the RNeasy Mini Kit (Qiagen, Germany). One microgram of RNA was digested with DNAse I (Fermentas, Canada) and reverse transcribed using the PrimeScript RT reagent kit (Takara, Japan), and cDNA was directly used as the template for PCR with SYBR Select Master Mix (Life Technologies, USA) using a Bio-Rad detection system (Bio-Rad, USA) and the primers listed in Table 2. The expression level of the housekeeping gene* rrsG* was used to normalize the expression levels of the target genes as described previously [23]. Both the WT strain and* ΔtolC* mutant Ct values were normalized to those of the housekeeping gene* rrsG* using the delta-delta Ct (ΔΔCt) method, with* ΔtolC* mutant strain Ct values representing the fold change relative to that of the WT strain, which was set at 1. Comparative qRT-PCR was used to determine the average expression from four replicate wells. The assays were repeated using RNAs harvested from independent cultures of each strain in triplicate.

### 2.10. Statistical Analyses

Statistical analyses of the data for biofilm production and gene expression were performed using SPSS 17.0 software (SPSS Inc., Chicago, IL, USA). The statistical significance of differences between the mutant and WT strain was calculated using the unpaired Student's* t*-test. Differences between groups were considered significant at a* P* value of < 0.05.

## 3. Results

### 3.1. Confirmation of the *ΔtolC* Mutant and Its Complement Strain

**Figure 1:**
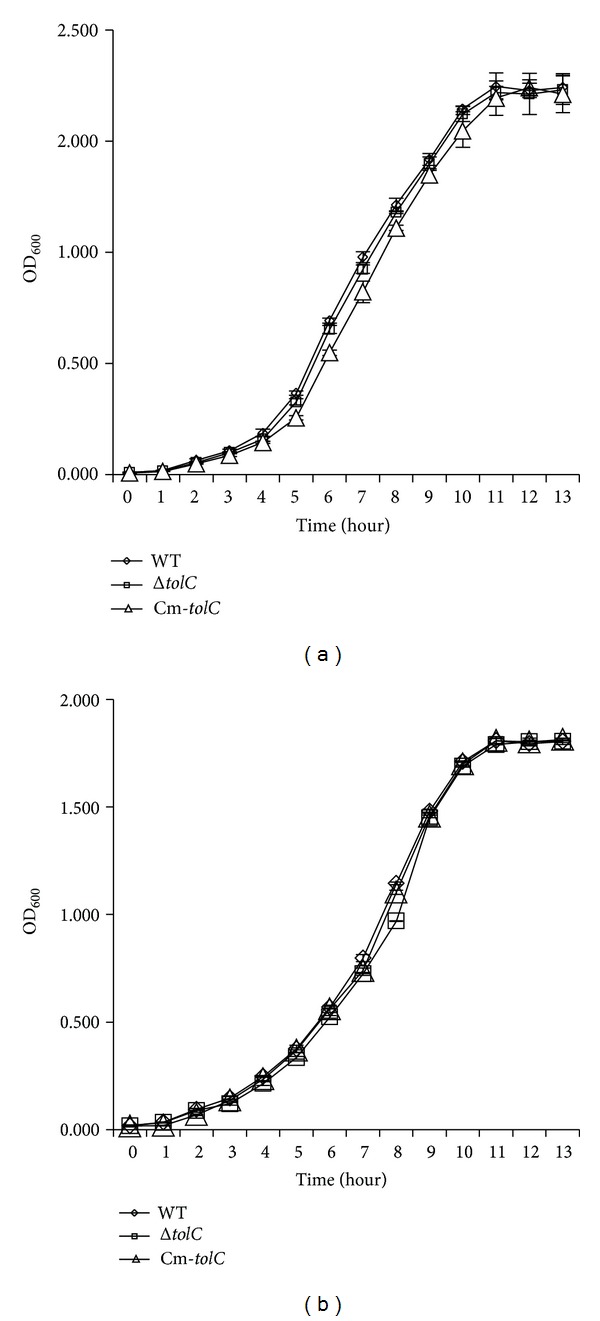
Growth kinetics of ExPEC wild-type (WT),* ΔtolC*, and Cm-*tolC* strains. WT (◊),* ΔtolC* strain (□), and Cm-*tolC* strain (△) grown in M9 media (a) or 1/2 M9 medium (b) at 28°C with shaking at 200 rpm. Bacterial strains were grown for 13 h, and the optical densities at 600 nm (OD_600_) were recorded hourly. Three independent cultures were tested in triplicate, and each point on the growth curve represents an average of nine readings.

The* ΔtolC* mutant and its complement strain constructed from ExPEC strain PPECC42 were confirmed by PCR, and the MICs of all strains to different antibiotics and toxic chemical agents were determined. Compared with the WT strain, the* tolC* mutant had a 4- to 64-fold increased susceptibility to eight agents, namely, amikacin, erythromycin, florfenicol, norfloxacin, chloramphenicol, tetracycline, ciprofloxacin, and SDS, but not to gentamycin, streptomycin, or ampicillin (Table 3). The complement strain containing plasmid* pHSG-tolC* restored the MDR to the levels of the WT strain. These results demonstrated the essential role of TolC in bacterial MDR as shown in some previous studies [12], indicating a correct* tolC* mutant was obtained. The growth kinetics of the WT and* ΔtolC* mutant experimental strains were detected in M9 or 1/2 M9 medium at 28°C with shaking. The results demonstrate that the* ΔtolC* mutant and its complement strain displayed similar growth kinetics to the WT strain, indicating that the* ΔtolC* mutant strain did not present a growth defect (Figure 1).

### 3.2. Osmolarity Affected the Capability of the *ΔtolC* Mutant but Not WT Strain to Form Biofilm

**Figure 2:**
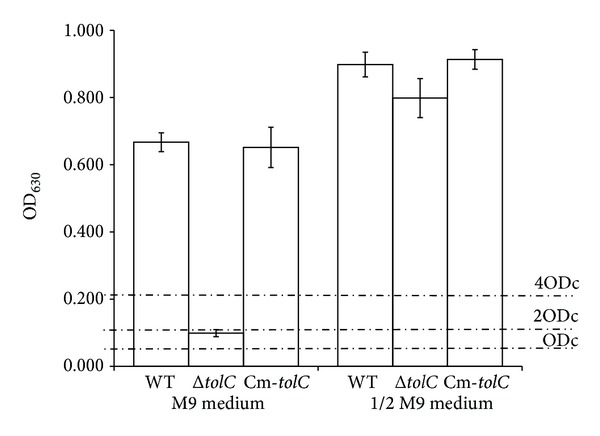
Crystal violet biofilm assay of ExPEC strains grown in M9 or 1/2 M9 media. Biofilms were formed in 96-well plates at 28°C for 5 days and quantified by measuring the OD at 630 nm (OD_630_) of dissolved crystal violet. Means and standard error of the mean values of OD_630_ values of 24 replicate wells from three independent cultures are shown. The cutoff OD (ODc) was defined as three standard deviations above the mean OD of the negative control. Biofilm production was classified as follows: OD < ODc = no biofilm production; ODc < OD < 2 × ODc = weak biofilm production; (2 × ODc) < OD < 4 × ODc = moderate biofilm production; and 4 × ODc < OD = strong biofilm production.

**Figure 3:**
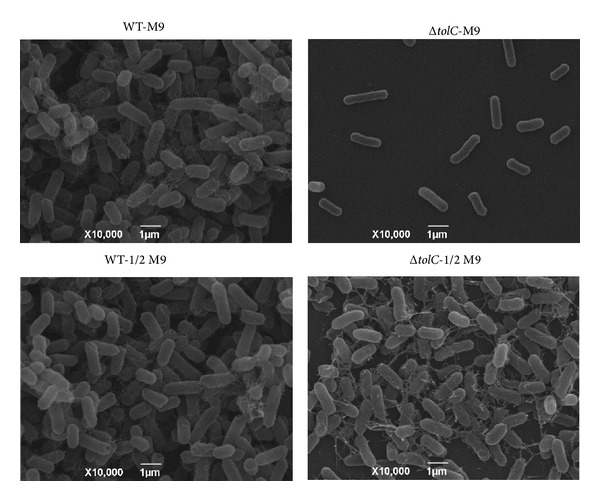
Scanning electron microscopy (SEM) images of the WT strain (left) and Δ*tolC* strain (right). All strains were incubated on glass coverslips at 28°C for 5 days in M9 medium (upper) or 1/2 M9 medium (lower) and viewed under 10,000x magnification.

Crystal violet biofilm assay demonstrated that the WT and complement strains cultured in 1 × M9 medium for 5 days at 28°C formed a strong biofilm (OD > 4 × ODc), whereas the* ΔtolC* mutant strain cultured under the same conditions formed a weak biofilm (ODc < OD < 2 × ODc; Figure 2). SEM of the biofilms revealed that the WT strain bacterial cells had a large number of extracellular fimbriae and formed a dense biofilm, whereas the* ΔtolC* mutant strain bacterial cells did not present extracellular fimbriae and failed to form a biofilm (Figure 3). These results indicate an important role for TolC in ExPEC biofilm formation.

As* E. coli* biofilm formation is regulated by osmolarity [19], the biofilm formation capabilities of the WT and* ΔtolC* strains cultured in a low-osmolarity 1/2 M9 medium were assessed using the crystal violet biofilm assay and SEM. Interestingly, all WT, complement, and* ΔtolC* mutant strains formed strong biofilms (OD > 4 × ODc; Figure 2). SEM of biofilms revealed that not only the WT strain but also the* ΔtolC* mutant strain bacterial cells had a large number of extracellular fimbriae and formed a dense biofilm (Figure 3), indicating that the* ΔtolC* mutation did not affect the ExPEC biofilm formation in a low-osmolarity medium.

**Figure 4:**
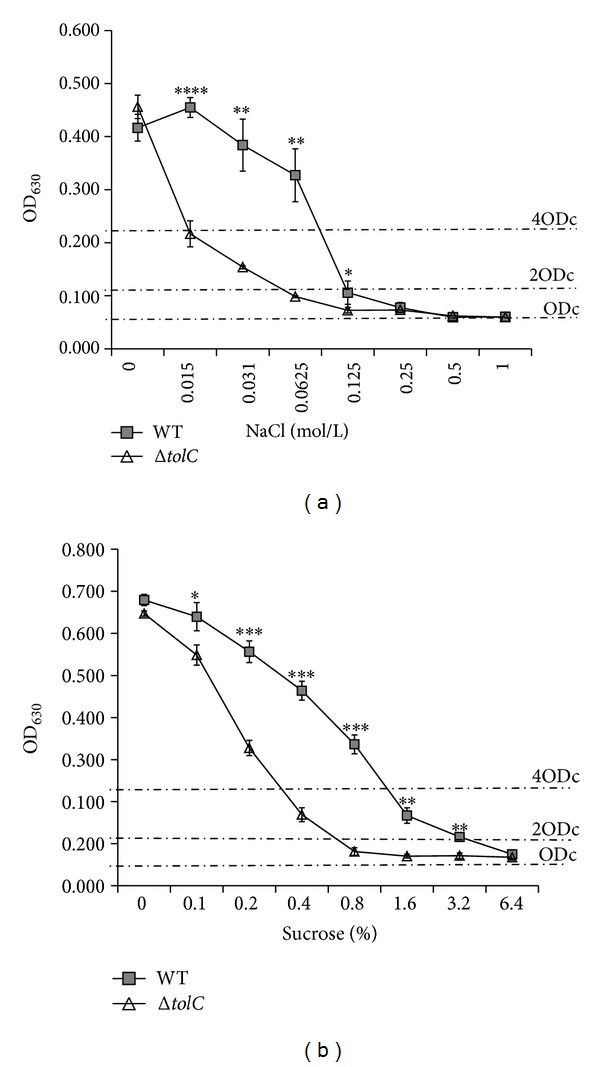
Effect of medium osmolarity on biofilm formation by the WT and Δ*tolC* strains as determined by crystal violet biofilm assay. Both strains were cultivated in 1/2 M9 medium supplemented with different concentrations of NaCl (a) or sucrose (b) at 28°C for 5 days. Biofilm formation was quantified, and the results are shown as means ± standard error of the mean. Significant differences between the WT and Δ*tolC* strains were determined using one-tailed unpaired Student's* t*-test. ∗*P* < 0.05; ∗∗*P* < 0.01; ∗∗∗*P* < 0.001; ∗∗∗∗*P* < 0.0001.

To further investigate whether the different biofilm formation capabilities of the* ΔtolC* mutant strain in the different media resulted from different osmolarities, we studied the biofilm formation capabilities of the WT and* ΔtolC* mutant strains grown in 1/2 M9 medium with different NaCl or sucrose concentrations. We found that biofilm formation of both strains reduced with an increase in the concentration of NaCl from 0.015 mol/L to 1 mol/L and sucrose from 0.1% to 6.4% (Figure 4). Although no significant difference in biofilm formation was observed between the WT strain and* ΔtolC* mutant cultivated in 1/2 M9 medium alone, the biofilm formation ability of the* ΔtolC* mutant was significantly reduced (*P* < 0.01) compared to that of the WT strain when the NaCl concentration was between 0.015 mol/L and 0.0625 mol/L or when the concentration of sucrose was between 0.2% and 3.2% in 1/2 M9 medium. The WT strain maintained a strong biofilm formation ability (OD > 4 × ODc) at NaCl concentrations up to 0.0625 mol/L or sucrose concentrations up to 0.8%, whereas the* ΔtolC* mutant merely exhibited a weak or absent biofilm formation ability (OD < 2 × ODc) at NaCl concentrations above 0.015 mol/L or sucrose concentrations above 0.4% (Figure 4). These results suggest that TolC plays an important role in tolerating different surrounding osmolarities for biofilm formation.

### 3.3. *ΔtolC* Mutation Reduced Curli Production in M9 Medium, but Not in 1/2 M9 Medium

**Figure 5:**
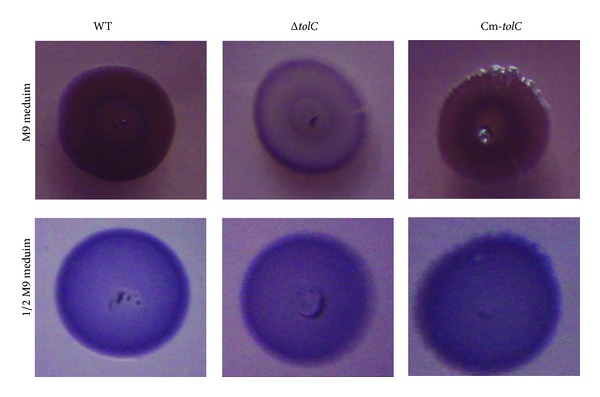
Effect of TolC on the production of curli fimbriae as determined by Congo red binding. All strains were cultivated on M9 agar plates (a) or 1/2 M9 agar plates (b) supplemented with Congo red at 28°C for 5 days. Visible Congo red binding was observed by the naked eye.

**Figure 6:**
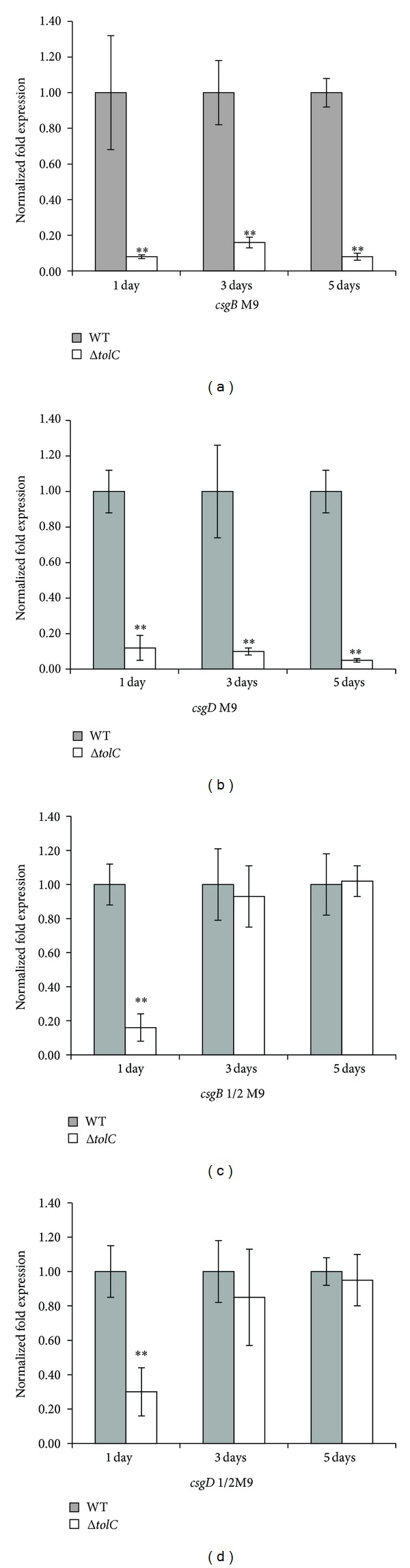
Effect of TolC on expression of genes related to curli biosynthesis. The* csgB* and* csgD* expression levels were detected by real-time qRT-PCR. The WT strain (dark columns) and Δ*tolC* mutant strain (light columns) were grown in M9 or 1/2 M9 medium at 28°C for 1, 3, or 5 days. Ct values were calculated relative to those of the housekeeping* rrsG* gene using the ΔΔCt method, with the Δ*tolC* mutant strain values representing the fold change relative to those of the WT strain, which were considered 1. Bars with different hatching patterns represent the average relative expression levels using RNAs from three independent bacterial cultures. Values are shown in means ± standard error of the mean. Statistically significant fold changes between the WT and Δ*tolC* mutant strains were determined using one-tailed unpaired Student's* t*-tests. ∗*P* < 0.05; ∗∗*P* < 0.01.

Curli is the major component of the extracellular matrix involved in* E. coli* biofilm formation [20, 21], and thus we next determined whether the influence of TolC on biofilm formation correlates with curli production using the Congo red assay and real-time qRT-PCR. The Congo red assay on M9-CR agar plates showed that the WT and complement strains developed a* bdar* morphotype and the* ΔtolC* mutant developed a* pdar* morphotype, suggesting that the* ΔtolC* mutation reduced curli production. However, all of the strains developed* bdar* morphotypes on 1/2 M9-CR agar plates, suggesting that the* ΔtolC* mutation had no obvious effect on the curli production in 1/2 M9 medium (Figure 5). Real-time qRT-PCR showed that, compared to the WT strain, the* ΔtolC* mutant strain cultured in M9 media for 1, 3, or 5 days had significantly decreased mRNA levels of* csgB* and* csgD* (*P* < 0.01). Although* csgB* and* csgD* expression were lower in the* ΔtolC* mutant strain than in the WT strain cultured in 1/2 M9 media for 1 day, no significant differences in* csgB* and* csgD* expression were observed between the two strains following culturing in 1/2 M9 medium for 3 or 5 days (Figure 6). These results indicate that the effect of osmolarity on* csgB* and* csgD* expression was temporarily restricted and that the function of TolC in curli production might be responding to different osmolarities.

## 4. Discussion

To elucidate whether TolC plays an essential role in tolerating different osmotic conditions in order to maintain the biofilm formation capability of ExPEC strains, we investigated the biofilm formation, curli production, and* csgB* and* csgD* expression of a* ΔtolC* mutant and its complement strains constructed in this study under different osmotic conditions. We found that the* ΔtolC* mutant strain grown on M9 medium lost the ability to form a biofilm and exhibited reduced curli production and diminished* csgB* and* csgD* expression.

We found that the* ΔtolC* mutant strain did not form a biofilm and showed reduced curli production in 1 × M9 medium but had a restored biofilm formation ability and comparable curli production in 1/2 M9 medium. The correlation of osmolarity with biofilm formation and curli production was further elucidated by increasing the medium osmolarity in the 1/2 M9 medium with NaCl or sucrose. Our findings indicate that TolC plays an important role in tolerating high osmolarity to maintain the biofilm formation capability in ExPEC strains. Biofilm formation by* E. coli* depends on media type, culture conditions, source, and phylogeny [32, 33]. Therefore, the WT strain formed a biofilm in M9 and 1/2 M9 medium at 28°C but not at 37°C or in LB medium at 28°C or 37°C, and the* ΔtolC* mutant strain only formed a biofilm in 1/2 M9 medium. These phenomena were in agreement with the notion that different environmental conditions, including growth medium and conditions, influence biofilm formation [34], and in particular, osmolarity correlates with curli production [19]. Hence, the* ΔtolC* mutant formed a biofilm in 1/2 M9 medium (a low-osmolarity medium) but not in M9 medium. These results suggest that TolC is important for ExPEC biofilm formation. In fact, we found that efflux pump inhibitors (carbonylcyanide-m-chlorophenylhydrazone (CCCP)) inhibited WT ExPEC strain biofilm formation in M9 medium (data not shown).

We found that the absence of TolC significantly reduced curli production. This finding agrees with those of some previous studies in* E. coli* and* S. typhimurium* [16–18]. In fact, curli fimbriae are the major components of the extracellular matrix involved in* E. coli* biofilm formation [20] and play important roles in the initial bacteria-surface interactions and subsequent bacteria-bacteria interactions and biofilm formation [20, 21, 35].

We found that the gene expression levels of* csgB* and* csgD* in the* ΔtolC* mutant strain were significantly lower than those in the WT strain within the first 24 hours of culture (*P* < 0.01), but restored in 1/2 M9 medium at 3 and 5 days. This* csgD* repression, resulting in low curli production in response to a high osmolarity by NaCl and sucrose, was mediated by CpxR protein and global regulatory protein H-NS [19]. Perhaps the* ΔtolC* mutation may alter the Cpx and/or H-NS pathway to affect the outer membrane integrity by regulating the expression of outer membrane proteins, such as OmpF and OmpC [9, 36]. The* ΔtolC* mutation may also cause accumulation of toxic metabolites inside the cell, which activates the BaeSR and CpxR stress response pathways [37, 38]. Therefore, these outcomes in turn influence the response to high osmolarity stress in the* ΔtolC* mutant strain. We speculate that, under a low osmolarity environment, the effect of the* ΔtolC* mutation might not be obvious, but, under a high-osmolarity environment, the ability of the* ΔtolC* mutant to respond to stress from the surrounding environment was compromised.

## 5. Conclusions

In conclusion, we found for the first time that TolC influences ExPEC biofilm formation and curli production by responding to a high-to-medium osmolarity change. Our findings provide insight into why ExPEC stains maintain their biofilm formation capability even under high osmotic stress and thus will contribute to the development of a treatment strategy targeting ExPEC biofilm formation. However, further study of how TolC copes with high osmotic stress to maintain the biofilm formation and curli production capabilities of ExPEC stains is necessary.

## Figures and Tables

**Table 1:** Bacterial strains and plasmids.

Strain or plasmid	Description	Source or reference
Strain		
ExPEC strain PPECC42	Wild-type (WT), porcine origin, Cm^S^	Lab stock
PPECC42Δ*tolC *	Mutant with a 158-bp fragment deleted from the whole ORF of the *tolC* gene in PPECC42, Cm^S^	This study
Cm*-tolC *	PPECC42Δ*tolC *strain containing plasmid pHSG-*tolC*, Cm^R^	This study
*χ*7213	Thi-1 thr-1 leuB6 fhuA21 lacY1 glnV44 Δ*asd*A4 recA1 RP4 2-Tc::Mu[*λ*pir] Km^R^	[22]
*χ*7213-Δ*tolC *	*χ*7213 containing suicide vector pRE112*ΔtolC *	This study
DH5*α*	F^−^, *φ*80d*lac*ZΔM15, Δ(*lacZYA-argF*)U169, *deoR*, *recA*1, *endA*1, *hsdR*17(r_k_^−^, m_k_^+^), *phoA*, *supE*44, *λ* ^−^, *thi-*1, *gyrA*96, *relA*1	Takara Bio
Plasmid		
*pRE112 *	oriT oriV *Δasd* Cm^R^ SacB, suicide vector	[22]
*pRE *Δ*tolC *	pRE112-inserted disrupted *tolC* gene in Kpn I and Sac I sites	This study
*pHSG396 *	ori lacZ Cm^R^	Takara Bio
*pHSG-tolC *	Cm^R^	This study

**Table 2:** Primers for PCR/RT-PCR.

Primer^a^	Sequence (5′-3′)	Application	Source or reference
*tolC*-P1	GCCGGTACCATGAAGAAATTGCTCC *Kpn* I	Mutant	This study
*tolC*-P2	CTATCGTCATAGGTTGCGTTTTTCGGCTTC	Mutant	This study
*tolC*-P3	GAAGCCGAAAAACGCAACCTATGACGATAGCAATATGGGCCAG	Mutant	This study
*tolC*-P4	TCCGAGCTCTCAGTTACGGAAAGGGT *Sac* I	Mutant	This study
*tolC*-clo-U	GGCGTCGACATGCAAATGAAGAAATTG *Sal * *Ι*	Complement	This study
*tolC*-clo-D	CGGGAATTCTCAGTTACGGAAAGGGTT *Eco*R *Ι*	Complement	This study
*tolC*-che-U	AACACGCTGCTGAAAGAA	Checking PCR	This study
*tolC*-che-D	ACGGTTTGTACGACGCTA	Checking PCR	This study
*rrsG*_ F_	TATTGCACAATGGGCGCAAG	qRT-PCR	[23]
*rrsG*_ R_	ACTTAACAAACCGCCTGCGT	qRT-PCR	[23]
*csgB*_ F_	CATAATTGGTCAAGCTGGGACTAA	qRT-PCR	[24]
*csgB*_ R_	GCAACAACCGCCAAAAGTTT	qRT-PCR	[24]
*csgD*_ F_	CCCGTACCGCGACATTG	qRT-PCR	[24]
*csgD*_ R_	ACGTTCTTGATCCTCCATGGA	qRT-PCR	[24]

^a^Subscripts F and R indicate forward primers and reverse primers.

**Table 3:** Antimicrobial susceptibility of the experimental strains.

	MIC (*μ*g mL^−1^)										
Strain	AMI	GEN	ERY	STR	AMP	FLO	NOR	CHL	TET	CIP	SDS
WT	16	>512	128	512	>512	128	512	64	256	256	≥1024
Δ*tolC *	4	512	4	512	>512	4	64	≤1	64	8	32
Cm-*tolC *	16	>512	128	>512	>512	64	512	N.D.	256	256	≥1024

AMI: amikacin; GEN: gentamycin; ERY: erythromycin; STR: streptomycin; AMP: ampicillin; FLO: florfenicol; NOR: norfloxacin; CHL: chloramphenicol; TET: tetracycline; CIP: ciprofloxacin; SDS: sodium dodecyl sulfonate.

N.D.: not determined because the Cm-*tolC* strain has plasmid *pHSG-tolC* with the gene resistant to chloramphenicol.
